# PKCδ Localization at the Membrane Increases Matrix Traction Force Dependent on PLCγ1/EGFR Signaling

**DOI:** 10.1371/journal.pone.0077434

**Published:** 2013-10-14

**Authors:** Joshua Jamison, Douglas Lauffenburger, James C.-H. Wang, Alan Wells

**Affiliations:** 1 Department of Pathology, McGowan Institute for Regenerative Medicine, University of Pittsburgh, Pittsburgh, Pennsylvania, United States of America; 2 Department of Orthopedic Surgery, McGowan Institute for Regenerative Medicine, University of Pittsburgh, Pittsburgh, Pennsylvania, United States of America; 3 Department of Biological Engineering, MIT, Cambridge, Massachusetts, United States of America; King's College London, United Kingdom

## Abstract

**Introduction:**

During wound healing, fibroblasts initially migrate into the wound bed and later contract the matrix. Relevant mediators of transcellular contractility revealed by systems analyses are protein kinase c delta/myosin light chain-2 (PKCδ/MLC-2). PKCδ is activated by growth factor-driven PLCγ1 hydrolysis of phosphoinositide bisphosphate (PIP_2_) hydrolysis when it becomes tranlocated to the membrane. This leads to MLC-2 phosphorylation that regulates myosin for contractility. Furthermore, PKCδ n-terminus mediates PKCδ localization to the membrane in relative proximity to PLCγ1 activity. However, the role this localization and the relationship to its activation and signaling of force is not well understood. Therefore, we investigated whether the membrane localization of PKCδ mediates the transcellular contractility of fibroblasts.

**Methods:**

To determine PKCδ activation in targeted membrane locations in mouse fibroblast cells (NR6-WT), two PKCδ constructs were generated; PKCδ-CaaX with farnesylation moiety targeting PKCδ to the membrane and PKCδ-SaaX a non-targeting control.

**Results:**

Increased mean cell force was observed before and during EGF stimulation in fibroblasts expressing membrane-targeted PKCδ (PKCδ-CaaX) when analyzed with 2D cell traction force and 3D compaction of collagen matrix. This effect was reduced in cells deficient in EGFR/PLCy1 signaling. In cells expressing non-membrane targeted PKCδ (PKCδ-SaaX), the cell force exerted outside the ECM (extracellular matrix) was less, but cell motility/speed/persistence was increased after EGF stimulation. Change in cell motility and increased force exertion was also preceded by change in cell morphology. Organization of actin stress fibers was also decreased as a result of increasing membrane targeting of PKCδ.

**Conclusion:**

From these results membrane tethering of PKCδ leads to increased force exertion on ECM. Furthermore, our data show PLCγ1 regulation of PKCδ, at least in part, drives transcellular contractility in fibroblasts.

## Introduction

Fibroblasts require time- and context-specific signaling for motility and contraction of the matrix. In cells that undergo motility/contractions, the filopodia/lamellipodium first extends and eventually adheres to the substrate/target. The cell body then impels towards the lamellipodium with subsequent rear retraction. Subsequent cell retraction is modulated through disruption of adhesions at the rear of the cell. Similar migration and contraction in the wound are stimulated by release of growth factors such as epidermal growth factor (EGF), VEGF, PDGF. Interestingly, as wound healing resolves, CXCR3 cytokines such as CXCL4, CXCL9, and CXCL10 are released, with their subsequent signaling preventing rear retraction. This signaling eventually leads to channeling the motile phenotype into amplified trans-cellular contractions required to contract to restore tensile strength to the tissue [1]. 

Components of the cell contractility and motility pathway have been identified. Growth factor and matrikine signaling through the epidermal growth factor receptor (EGFR) initiates motility via phosphorylation and activation of PLCy1 at the membrane [2]. Activated PLCy1 then catalyzes the hydrolysis of PIP_2_ primarily at the leading edge and generates diacylglycerol (DAG) and IP3 [3,4]. Increased levels of DAG at the leading edge [5] synergizes the effect of PKCδ localization to the membrane[6]. DAG subsequently stabilizes the activation of PKCδ through direct binding of its N-terminal C1 domain [7–9]. Furthermore, PKCδ localization behind the leading edge allows it to propel the cell body towards the extended lamellipodium and also mediate isometric force concomitant with motility [10]. 

We previously showed that the EGFR-induced activation of PKCδ modulates force through an intermediate kinase, myosin light chain kinase (MLCK). MLCK can directly phosphorylate (myosin-light-chain) MLC to induce cellular contractions [11]. Furthermore, reduced activation of PLCy1 delayed subsequent activation of PKCδ and downstream MLC2. This caused inefficient contractions by the cells compared to normal PLCy1 signaling [11]. These data indicate that EGFR triggers contractile responses efficiently and quickly through PLCy1/PKCδ pathway. However, how the spatial localization of PKCδ to upstream modulators mediates force signaling has not been demonstrated. Therefore, PKCδ regulation of contraction and force distribution was investigated through its membrane translocation to PLCy1 activity. 

## Results

### Membrane targeting of PKCδ increases extracellular force on substratum

**Figure 1:**
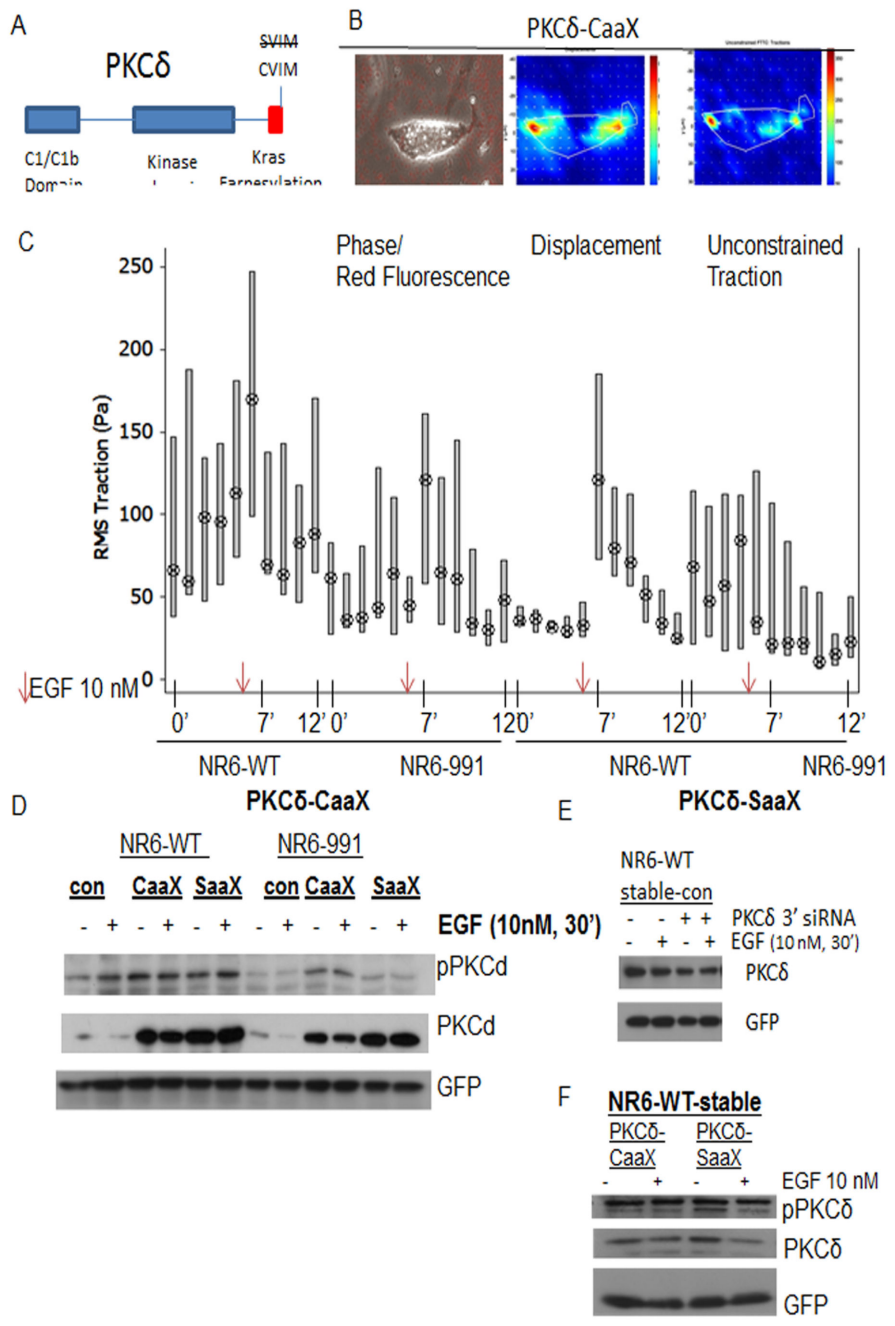
Membrane targeted PKCδ increases force of isometric contractions through EGFR/ PLCγ1 signaling. (*Cell* *Traction* *Force* *Microscopy*). a) A schematic of PKCδ showing the kras farnesylation motif at the c-terminus of the protein. Membrane targeted PKCδ (PKCδ-CaaX)is represented with the CVIM domain and non-membrane targeted (PKCδ-SaaX) is represented by SVIM. b,c)PKCδ-CaaX cells were placed on (0.5µm red beads) were prepared with 100 µg collagen cross-linked to PAG/beads. b) Cell traction was extrapolated through bead displacement as the cells exerted force. All forces exerted onto the substratum of each cell by bead displacement were computationally measured and analyzed using the software MatLab environment [26–28]. Unconstrained traction is force exerted by the cells in kPa that is derived from bead displacement on 3kPa PAG/bead gel and described previously in (25-27). Traction force output with unconstrained traction and absolute bead displacement from data extrapolation was gathered from all groups for each individual cell. Colorimetric indicators displays red as the most intense in traction force and dark blue displays minimal traction force. Images of cells were taken at 20X objective magnification. c) Boxplot of individual cell constrained force measurements between 25^th^ and 75^th^ percentile. Collective statistical analysis via Student’s T Test was performed between NR6-WT PKCδ-CaaX and NR6-991-PKCδ-Caax after EGF treatment at p = 6.87821e-09). As indicated in results and methods, NR6-WT cell lines contain full length EGFR and NR6-991 cell lines contain truncated EGFR that is deficient in PLCy1 signaling. d) Immunoblot analysis of cells transiently transfected with (PKCδ-C/SaaX) and 50 µM of siRNA of mouse PKCδ siRNA into NR6-WT and NR6-991 fibroblast were then incubated in quiescent media overnight and treated with EGF for 1 hour prior to cell lysis. Western blot analysis of cell lysates was performed. GFP that is expressed with the vector was utilized as control for protein levels. e) Lysates of siRNA knockdown of endogenous PKCδ of fibroblasts is represented in immunoblot. The non-linked GFP on the same vector were utilized for loading control. f) Immunoblot of cell lysates with stably transfected PKCδ-CaaX and PKCδ-SaaX. Non-linked GFP protein levels were utilized for loading control.

To investigate whether membrane targeting is sufficient to initiate trans-cellular contractility, PKCδ was directed to the membrane by splicing the farnesylation site of K-ras to the C-terminus [12](Figure 1a). These PKCδ constructs in a bicistronic vector expressing GFP were then stably transfected into mouse fibroblast cells with either reconstituted full length EGFR (NR6-WT) or a truncated EGFR that fails to activate PLCγ (NR6-991). To specifically investigate how membrane targeted PKCδ affects individual cell force that is exerted onto the substratum, contractility was assessed utilizing cell traction force microscopy. 

 Cells expressing PKCδ-CaaX exerted increased contraction of the substratum. This increased force was mainly localized at the front or rear of the cells with the cells appearing generally non-motile (Figure 1b). Furthermore, PKCδ-CaaX expressing cells also exerted increased tension at non-peripheral parts of the cell possibly due to the ubiquitous expression of PKCδ localization at the membrane. The cells responded to EGF quickly with concerted forces being exerted on to the substratum before and after EGF treatment compared to PKCδ-SaaX (Figure 1c). 

PKCδ-CaaX localization in the cell would position it closer to PLCγ1 activity (hydrolysis of PIP2) after EGFR stimulation [13] or simply move it to be activated constitutively by phosphatidyl serine on the inner membrane. To determine whether PLCγ1 signaling was required for force generation through membrane targeted PKCδ, cells that fail to activate PLCy1 signaling upon EGF exposure (NR6-991), were investigated for cell force generation. NR6-991 cells could not exert as much force as NR6-WT (Figure 1c). Molecular signaling of PLCy1 was further investigated in membrane-targeted PKCδ expressing cells. Decreased phosphorylation of PKCδ in response to EGF was observed in cells challenged with PLCy1 deficient signaling, suggesting full PKCδ effects are PLCy1 mediated (Figure 1d). In addition, knockdown of endogenous PKCδ and similar levels of protein expression from constructs were confirmed in stable cell lines (Figure 1e, Figure 1f). These results reinforce the rationale that EGFR stimulation of PLCy1 is key to PKCδ mediated fibroblast contractility. 

### Membrane-targeted PKCδ localizes to cell membrane to induce force signaling

**Figure 2:**
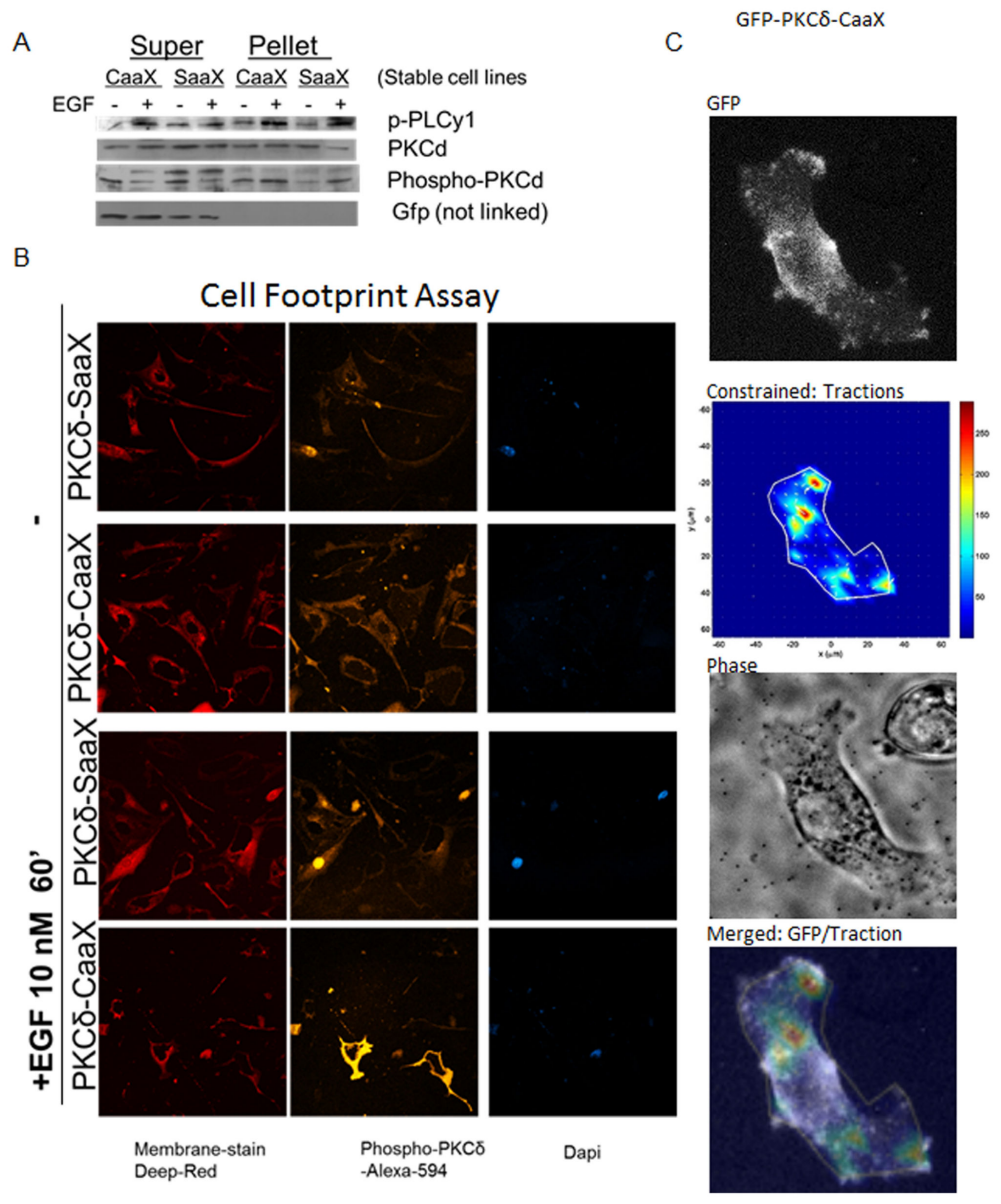
Membrane-targeted PKCδ at the membrane maps with force distribution. a) Stably transfected PKCδ-CaaX and PKCδ-SaaX NR6-WT cells were stimulated with 10 nM of EGF in quiescent media. After hypotonic fractionation, lysates were divided either into supernatant, which contains cytoplasmic proteins or pellet which contains membrane-linked proteins. Lysates were subjected to SDS-PAGE and immunoblotted for indicated proteins. GFP was utilized as a negative control for cytoplasm contamination in membrane fractions. b) Stably transfected PKCδ-CaaX and PKCδ-SaaX NR6-WT cells were stimulated with 10 nM of EGF in quiescent media for 60 minutes prior to fixation. Footprints were collected as described in methods and were immunostained for activated PKCδ. Images were then taken of footprints with confocal microscopy at 40x objective magnification. DAPI was utilized as a negative control for the presence of the nucleus, which is removed in the process of retaining the bottom membrane only attached to the substrate. The Deep-Red membrane stain was utilized as a positive control for membranes. c) DNA constructs with GFP linked to PKCδ-CaaX and PKCδ-SaaX were transfected in NR6-WT. Cells were then plated onto PAG/beads substrate as described previously in (Figure 1) in the presence of culturing media. Images of cells were taken at 20x objective every 10 minutes as localization of PKCδ was observed and force was extrapolated from bead displacement represented in constrained traction force indicated in colorimetric graph. In colorimetric graph, red represents high traction force and blue represents low/no traction force. Merged images of constrained traction force and GFP PKCδ localization is indicated. Red represents strong force and white represent PKCδ localization.

 PKCδ membrane translocation is essential to regulation of its activity. To determine how increased membrane targeting affects PKCδ activation, membrane and cytosolic fractions of PKCδ were analyzed comparing the two constructs in stably transfected cell lines. From these data, there was increased total PKCδ in the membranes of PKCδ-CaaX stably transfected cells compared to PKCδ-SaaX expressing cells. EGF stimulation activated both PKCδ-CaaX and PKCδ-SaaX at membrane indicated by increased phosphorylated PKCδ fractions (Figure 2a). In addition, depletion of cytosolic fractions of activated PKCδ during EGF stimulation was also observed, confirming net translocation of PKCδ as opposed to de novo synthesis. Although activated PKCδ-SaaX increased at the membrane during EGF stimulation as expected, these data also indicate that activated PKCδ-CaaX was increased in membrane fractions even prior to EGF treatment. This localization prior to EGF stimulation was intended and partially obviated the need for stimulation by EGF. 

 In addition, this increase in phosphorylated PKCδ localization to the membrane was further tested in specific cells through a ‘cell footprint’ assay. Similarly, activated PKCδ localized to the membrane prior to EGF stimulation (Figure 2b). After EGF stimulation, the activated PKCδ was found mainly to be membrane-targeted in comparison with a decrease in non-membrane-targeted fractions. These data suggest that membrane targeting increases PKCδ localization to the membrane for activation in response to EGF and membrane targeting in itself partially acts as a stimulus. 

 Localization of PKCδ and its impact on force transduction was further investigated by visualizing PKCδ through tagging the membrane targeted PKCδ with GFP (Movie S1). Cells transfected with this construct were analyzed by cell traction force microscopy. Cells that expressed PKCδ-CaaX increased cortical tension close to the peripheries of the cell whereas the non-membrane targeted PKCδ localized throughout the cytoplasm with little effect in morphology (Figure 2c). We furthermore found that PKCδ localization correlated with specific force being exerted onto the substratum prior and during PKCδ localization. These forces were exerted primarily behind the leading edge, along with some random specific non-peripheral force transduction. These data suggest PKCδ localization is directly associated to the distribution of force to the cells. 

### Membrane targeted PKCδ displays increased contraction of collagen gel compared to non-targeted PKCδ expressing cells

**Figure 3:**
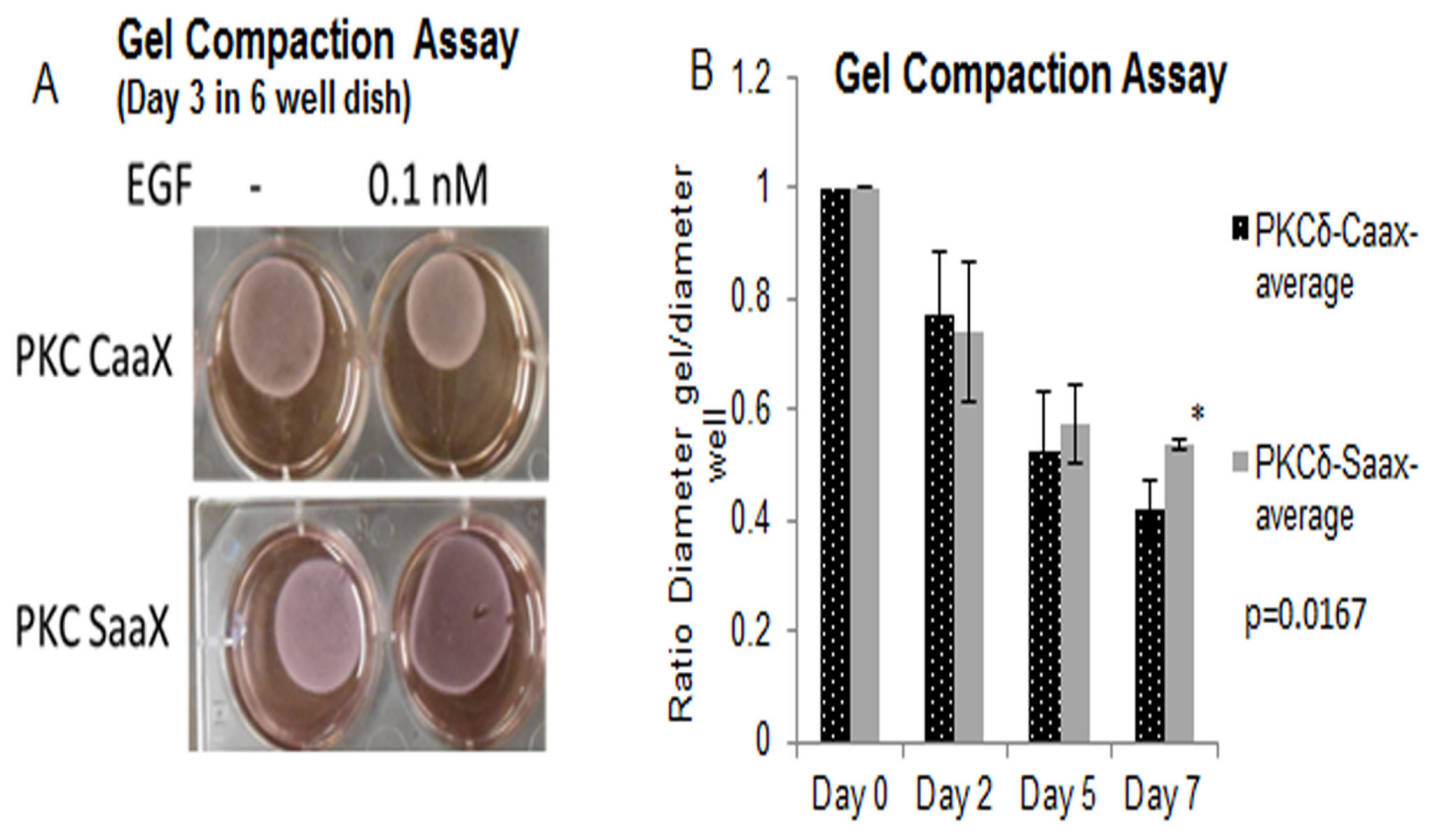
Gel compaction is increased in cells expressing membrane-targeted PKCδ. a,b) Stably transfected PKCδ-CaaX and PKCδ-SaaX NR6-WT cells were incubated in 1 mg/ml of polymerized collagen. Collagen gel and cells were incubated with growth factor for indicated time points and compaction was observed by visually measuring the size of collagen gel relative to well size. a) Picture of gels were taken. b) Ratio of gel size to well was calculated by image J line scan parameter of no EGF treated sample and analysis of multiple gel compaction assays (n=3) was performed as previously described and ratios were analyzed by Student-T-test p=.006.

 Cell motility and isometric cell force both contribute to the eventual compaction of both wound bed collagen/ECM and artificial collagen ECM [1]. To further investigate whether membrane targeted PKCδ causes increased force in a 3D ECM, the collective ability of cells to compact a collagen gel over time was investigated utilizing a gel compaction assay. Cells expressing PKCδ-CaaX mediated increased gel compaction compared to non-targeted PKCδ-SaaX (Figure 3), at which became significant at longer time periods (Figure 3b). These data suggest membrane targeted PKCδ predisposed cells to increased signaling for compaction which led to increased compaction of collagen gels compared to PKCδ-SaaX. 

### Non-membrane targeted PKCδ presents increased cell motility

**Figure 4:**
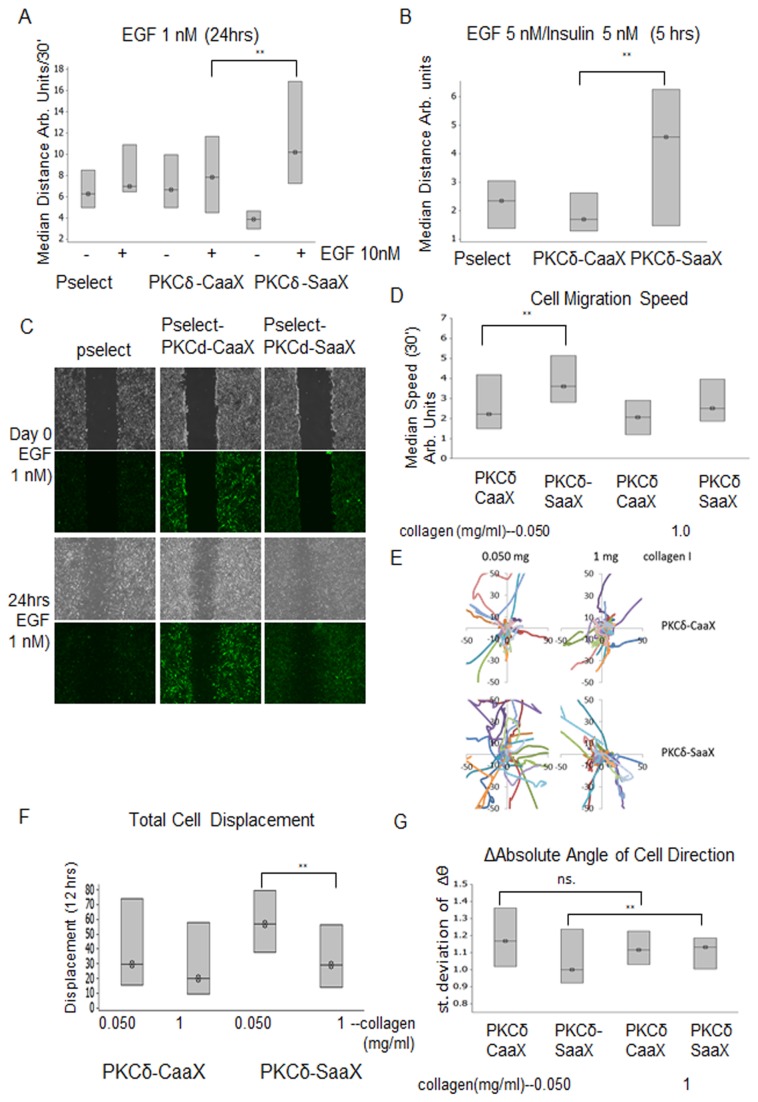
Cell motility is restricted by membrane targeting of PKCδ. a). Live cell images of stably transfected PKCδ-CaaX and PKCδ-SaaX NR6-WT (EGFR) cells with endogenous knockdown of PKCδ were taken every 30 minutes with and without 1 nM of EGF in quiescent media for 24 hour time period. Cell motility was analyzed by metamorph software. (Two-Sample-t-test was utilized to evaluate significance p<.05, n>11). b) Live cell images of stably transfected PKCδ-CaaX and PKCδ-SaaX NR6-WT (EGFR) cells with endogenous knockdown of PKCδ were taken every 15 minutes stimulated with 5nM of EGF and 5 nM of insulin for 5 hours. Average individual cell speed/path was analyzed at 15 minute intervals for a total duration of 5 hours with metamorph software. (Two-Sample-T-test was utilized to evaluate significance (p < 0.05, n >11). c) Stably transfected PKCδ-CaaX and PKCδ-SaaX NR6-WT (EGFR) were grown in a 6 well plate to 90% confluency prior to scratching with rubber policeman. Cells were treated with 1 nM of EGF in quiescent media and images of scratch were taken at 4X objective magnification. d,e,f,g) Stably transfected PKCδ-CaaX and PKCδ-SaaX NR6-WT (EGFR) *cells* *were* *grown* *to 90% confluency prior to plating on collagen I coated plate. Cells were then allowed to adhere overnight and then transfected with 50uM of* PKCδ specific siRNA and stimulated with 1 nM EGF. Cells were then imaged for 12 hours with 30 minute intervals and average individual cell speed (d) /path (e) /persistence (absolute displacement from origin)(f) was analyzed with metamorph software. (g) Persistence was analyzed utilizing the median of absolute angle standard deviation relative to control.

Our earlier systems biology analysis of motility signaling highlighted the adhesion to contractility ratio as key to motility [14], along with the ability to labilize or turnover adhesions. Thus, we sought to determine the effect of a lower level but tonic activation of the contractility pathway as driven by membrane-targeted PKCδ (Figure 4). Live cell imaging of stably transfected PKCδ-C/SaaX cells were observed on a collagen coated plastic substratum with knockdown of endogenous PKCδ. Analysis of random cell motility during live cell imaging showed that cells expressing PKCδ-CaaX moved faster in an unstimulated mode. Following EGF stimulation, PKCδ-SaaX moved faster than PKCδ-CaaX (Figure 4a). To further determine the extent of collective migration, a scratch wound healing assay was utilized. PKCδ-SaaX was found to move farther into the scratch compared to PKCδ-CaaX (Figure 4d). These findings are consistent with increased cell adhesion leading to decreased cell motility. Our approach to utilize forced membrane targeting of PKCδ does cause increased force into adhesions unto the substratum that subsequently results in decreased cell motility. 

To investigate whether membrane targeted PKCδ involves increased force exertion onto the substratum during motility, cells were challenged to migrate on an adhesive substrate. The increased adhesion would increase PKCδ/MLC activation while causing decreased cell speed in normal fibroblasts [14]. Furthermore, increased intracellular force during active cell motility would be able to overcome the effect of an increased adhesive substrate. From our results membrane targeted PKCδ remained at the same level of persistence at low to high collagen content, with slightly decreased cell motility (Figure 4e). Cells expressing the non-targeted PKCδ-SaaX were observed to have more persistent paths compared to PKCδ-CaaX with increased cell speed with low adhesive substratum (Figure 4f, 4e). When challenged with an adhesive substrate, non-membrane targeted PKCδ had reduced cell speed on the adhesive substrate with decreased motility persistence compared to PKCδ-CaaX indicated in (Figure 4f, Figure 4g, Figure 4h). These data suggest in membrane targeted PKCδ expressing cells decreased cell speed may be due to increased force to the substratum at a level to overcome the effects of a very adhesive substrate. 

### Membrane targeting of PKCδ activates stress fibers and leads to morphological changes independent of growth factor exposure

**Figure 5:**
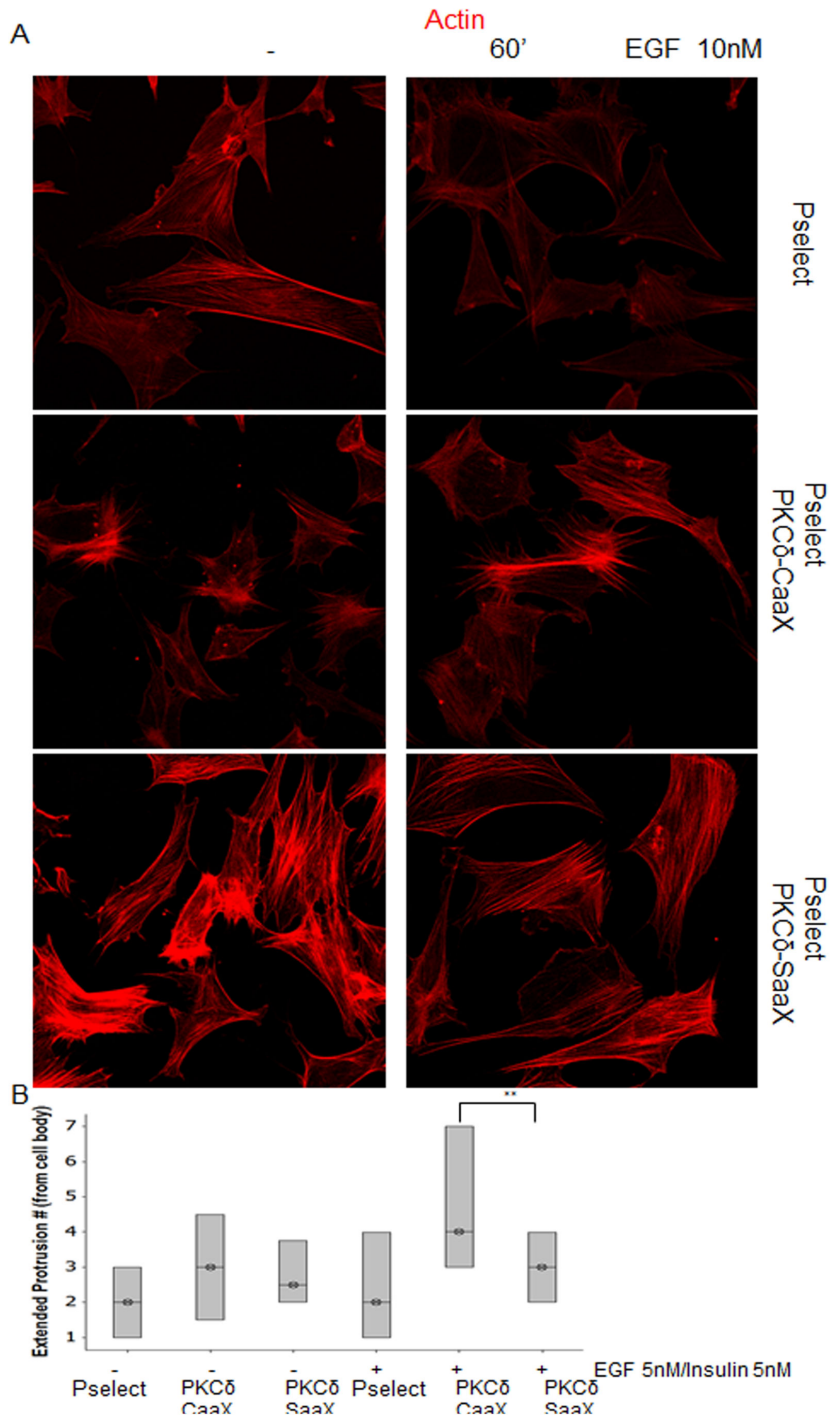
Membrane-targeting of PKCδ cytoskeletal structure of cells, without altering cell signaling of EGFR/ PLCγ1 / PKCδ pathway. a) PKCδ CaaX/SaaX stably transfected cells were incubated in quiescent media prior to treatment of EGF (10 nM) for 60’ and were fixed with 4% formaldehyde solution, permeabilized, and stained for phalloidin. Fluorescent images were taken utilizing confocal microscopy at mid z-stack. b) Cells were grown and transfected as previously described in (Figure 3a). Extended protrusions from cell body were manually counted after 5 hours and graphed, (p < 0.05, n>11).

Cell morphology change precedes growth factor stimulated cell motility [3]. To investigate how cells are affected by force signaling, stable cell lines were analyzed for stress fiber organization. To further investigate cytoskeletal tension resulting from PKCδ localization to the membrane, stress fibers of stably transfected cells were visualized by rhodamine-labeled phalloidin. Fibroblasts with PKCδ-CaaX demonstrated disorganized stress fibers, even prior to EGF stimulation. In contrast, more organized and pronounced stress fibers were observed in PKCδ-SaaX expressing cells (Figure 5a). These results show that distribution of force to the ECM disrupts stress fibers to the cortex and cell body. 

 To investigate how increased membrane targeting translated to cell morphology, we induced increased kras farnesylation by adding insulin in combination with EGF. This stimulus would increase PKCδ membrane targeting. We found that increasing membrane targeting caused increased protrusions (Figure 5b, Movie S2 and Movie S3). Of interest, PKCδ-SaaX correlated with fewer protrusions as normal localization of activated PKCδ is cell front limited. However, this increase in protrusions only occurred with this stimulus. EGF stimulation alone did not cause these results. These data suggest decreased cortical stress fibers allow for the plasticity of membrane targeted PKCδ to exert protrusions to the ECM.

## Discussion

 We have shown that increasing PKCδ translocation by membrane tethering redistributed force signaling outward to the ECM that is partially PLCγ1 dependent (Figure 1c). In addition to isometric force exerted under the cell to the substratum, force that is exerted on the cell body is also a significant portion of cellular contraction. Force on to the cell body can be indirectly measured through live cell motility[1]. Membrane targeted PKCδ caused a shift in cellular force from the cell body to the ECM. As a result, decreased cell speed was observed suggesting that the increased force ‘froze’ the adhesions (Figure 4a-d). In non-membrane targeted PKCδ expressing cells, distribution of force was manifested by increased cell speed compared to membrane targeted PKCδ expressing cells. As migration involves a cycle of de-adhesion, these cells also presented a reduced net extracellular force to the ECM (Figures 1, 3). As increased restrictive forces to the cells occurred during motility, cells expressing membrane-targeted PKCδ were more resilient to the effects of an adhesive substrate as determined through persistence measurements (Figure 4e-h). These data indicate that slightly shifting the dynamics of PKCδ localization shifts signaling of force distribution. This is a very specific effect, since cells were not manipulated with any other regulators of the cytoskeleton. 

 Interestingly, the difference between PKCδ-CaaX and PKCδ-SaaX were negligible in total downstream signaling to MLC-2. This implies that signaling of the proteins are the same with similar levels of expression (Figure 1e). However, localization of exerted force is the key determinant, and highlights the need to examine signaling cascades in subcellular compartments. Furthermore, from our studies only PKCδ-CaaX localizes to the membrane with increased activation of PKCδ. This furthermore correlates with cellular force distribution to the ECM (Figure 2). Considering their similarity, the differences in cellular responses are due to the intended difference in localization dynamics and resultant activation. 

 Force distribution to the ECM and force distribution to the cell body are both simultaneously and reciprocally being applied. As cells adhere to the ECM and actively migrate on a 2D substratum, forces emitted by these two actions are required by the cell for active motility. In a 3D-context, such as in a gel compaction assay, force exertion from the cells and forces applied to the cells are collectively systemic [15]. Each cell integrates its force into the system with increased plasticity and synergism impacting contractions of the ECM [16]. From this study, increased ECM compaction was observed as a result of signaling of force through membrane-targeted PKCδ (Figure 3). Signaling through growth factors and cytokines integrate cellular responses to coordinate systemic contraction of a wounded matrix. This study primarily focused on EGF signaling, since it is an essential growth factor for motility during wound healing. Downstream of EGFR signaling, EGFR stimulation of PIP_2_ hydrolysis impacts divergent regulation of motility and contraction. Although, PKCδ regulation is downstream of PIP_2_ hydrolysis, it has also been found to activate m-Calpain through direct binding [12]. As PIP_2_ is being hydrolyzed at the leading edge, the rear of the cell retains PIP_2_ levels where it aids in activation of m-Calpain to cleave rear adhesions [12,17]. This further supports the subcellular directionality of EGFR mediated PIP2 hydrolysis, and reinforcing the concept that spatial localizations of signaling nexi are important for productive motility [3]. Among context-specific functions of fibroblasts in wound healing, the mechanics for ECM remodeling [1] is regulated by both motility and isometric contraction critical for remodeling of the compacting ECM [18–20]. These factors in combination with the transient release of both growth factor and cytokine antagonist regulate the dynamic and synchronous relationship of how fibroblasts mediate this motility and contraction of the wound. If not properly regulated, this fine-tuned system that is mediated by durataxis and chemotaxis, may shift to exacerbate the healing tissue into fibrosis or fibroplasia [21,22]. 

## Materials and Methods

### Cell Culture

NR6-WT and NR6-991 cell lines were established previously from parental Swiss mouse 3T3 variant fibroblasts that lack endogenous EGFR as an original gift from Dr. Harvey Herschman [23]. These cells were cultured in minimal essential media (MEM) supplemented with 2 mM L-glutamine, 1 mM sodium pyruvate, 0.1 mM MEM, nonessential amino acids, penicillin (100 units/ml), streptomycin (100 µg/ml), and G418 (350 µg/ml) for continual selection of EGFR. Subconfluent (< 75%) cultures were split every 4 days using 0.25% trypsin/0.25 mM EDTA in MEM to dislodge cells from culture and washed further with MEM before seeding.

### Plasmid Construction and transfection

PKCδ constructs were established from human-derived Hs68 fibroblast cell line, in which the Kras *mouse* sequence (CaaX for farnesylation; SAAX for control) was spliced to the carboxyl-terminus of PKCδ in 3 rounds of PCR amplification. Recombinant PKCδ was then ligated into the p-select-GFP-zeocin plasmid (Invitrogen) using the *Bam*H1 and *Nhe*1 sites. Stable transfection was performed with 4µg of each plasmid (PKCδ-caaX or PKCδ-saax) and lipofectamine according to the manufacturers’ protocol. Following transfection, cells were grown and split in MEM containing 300µg/mL zeocin (Invitrogen). 

In addition to PKCδ transfection, 50 µM mouse siRNA targeting the PKCδ 3’ UTR was also transfected into transient and stably transfected cells. The primers used for siRNA synthesis were 5’-AACACAUCACCAGUCUCCUACAUGCUU-3’ and 3’-TTGUGUAGUGGUCAGAGGAUGUACG-5’ respectively. This sequence was designed using the online software from Integrated DNA Technologies. 

### Cell Traction Force Measurements

Cell Traction Force Microscopy protocol was performed as previously described [24]. Briefly, 6 well glass bottom plates (Mattek) were first activated by treatment with 0.1M sodium hydroxide for 1 day and allowing to air-dry overnight. The next day approximately 2 drops of 3-aminopropyltrimethoxysilane was added to each well followed by washing with de-ionized water, incubation in 0.5% glutaraledehyde for 1 hr and finally air dry. After activation, the first layer of gel was made with 11µL polyacrylamide (5% acrylamide and 0.1% bisacrylamide), 20 µL of 10% ammonium persulfate and 2µL TEMED and poured on the activated glass bottom plates. A circular glass coverslip was then placed on top of the solution. After polymerization, the glass coverslip was removed and a second layer of gel as described previously but with 0.5 µM fluorescently labeled beads was poured on top of the first layer and the gel was incubated overnight in water with a glass coverslip placed on top. Collagen was then crosslinked to the gel by adding sulfo-SANPAH on the top followed by exposure to UV-radiation. After washing 4 times with PBS, collagen I (150 µg/ml, BD Bioscience) was added on the gel and allowed to crosslink with sulfo-SANPAH overnight. 

 Prior to plating fibroblasts onto the polyacrylamide gel, cells were transfected with 50 µM PKCδ siRNA and incubated overnight in MEM. Transfected cells were then detached by trypsinization and added to polyacrylamide gels in quiescent media and allowed to adhere for at least 5 hrs. Live cell images were taken at indicated time points with a 20X objective, and bead displacements and force were computed using the MatLab programming software as previously described [24]. 

### Primary antibodies and reagents

For immunoblotting and immunostaining the following antibodies were used at a dilution of 1:1000: Anti-PKCδ antibody (BD Biosciences), anti-phospho-PKCδ (S643 PKC delta/S676 PKC-theta), anti-ppMLC-2 (S18/19), and anti-MLC-2 (Cell Signaling), and anti-GFP-FL (SantuCruz). For actin staining, phallodin-conjugated to Alexa-568 (Invitrogen) was added at 1:40 dilution. 

### Gel compaction assay

Stably transfected NR6-WT fibroblasts were grown in polymerized collagen I using previously described methods with modifications [11]. Stably transfected PKCδ-CaaX and PKCδ-SaaX expressing cells were cultured to subconfluence and harvested using 0.25% trypsin/EDTA. Cells were then resuspended in MEM, diluted to 1 x 10^6^ cells/mL and centrifuged at 1000 rpm for 5 min. Fibroblasts were then resuspended in quiescent media containing 1mg/mL bovine serum albumin and EGF at various concentrations. Neutralized collagen solution (1mg/ml collagen/media-pH-7) was immediately mixed with fibroblast solution and allowed to polymerize for 1 hr at 37°C. After polymerization, collagen was released from the sides of the wells by a small pipette tip. Compaction was determined by decrease in the size of the collagen gel which was documented as images. Quantification of the images was performed by line scan procedure using the image J software. From this analysis we obtained the ratio of the diameter of standardized well to the diameter of collagen gel, as diameter was preferred over area of compaction due to little difference in experimental outcome and significance. 

### Motility Measurements

Stably transfected cells were grown as previously described and plated onto collagen coated plates. Afterwards, cells were transfected with 50uM siRNA for 24 hours prior to imaging. Cells were then incubated in quiescent media overnight and treated with 1 nM of EGF for 24 hours in In live imaging chamber at (5% CO_2_, 4% O_2_). Cells treated with insulin were incubated at 5nM EGF and 5 nM insulin to increase membrane targeting at 5 hour time period. Cell speed was tracked utilizing metamorph software utilizing the track object function. Individual cells were highlighted and the software computationally tracked cell movement as cell displacement in each frame along xy coordinates. 

### Hypotonic Subcellular Fractionation

Subcellular fractionation was described previously [25]. Briefly, stably transfected cells were grown to subconfluency prior to quiescence media incubation overnight. Cells were then treated with 10 nM of EGF for 60 minutes. Cells were then scraped at 4C with rubber policeman and lysed with hypotonic buffer (10mM HEPES pH 7.4, 1.5mM MgCl_2_, 10 mM KCl, 0.2 mMphenylmethylsulfonyl fluoride, 0.5 mM dithiothreitol). Cell lysates at 0.5 mL were then homogenized on ice further with dounce at the rate of 40 strokes. Unhomogenized cellular debris was removed by centrifugation at 1,000 x g for 5 minutes. Supernatant was then subjected to ultra-centrifugation at 100,000 x g for 1 hour at 4C. Supernatant and pellet were then separated and extracted with 1X sodium dodecyl sulfate (SDS) sample loading buffer.

### Cell Footprinting

The dorsal part of the cell was removed as described previously [5,12]. Stably transfected cells were plated onto collagen coated (50ug/mL) glass coverslip prior to incubation of quiescent media. Cells were then stimulated with 10 nM of EGF for 60 minutes prior to cell footprint isolation. In addition, a.ll isolation solutions were incubated at 4°C prior to use. Fibroblasts were washed with morpholineethanesulfonic acid-buffered saline (MBS; 20 mM morpholineethanesulfonic acid [pH 5.5], 135 mM NaCl, 0.5 mM CaCl_2_, 1 mM MgCl_2_). Cells were then coated with a 1% solution of cationic colloidal silica (silica prepared as a 30% stock colloid). (Cationic colloidal silica was obtained by written request from Donna Beer Stolz, University of Pittsburgh, Pittsburgh, PA.) Repeat of wash with MBS was done prior to coating cells with 1% polyacrylic acid (Sigma Aldrich) in MBS. Polyacrylic coat was removed with another wash of MBS. Cells were then swelled for 10 minutes with hypotonic lysis buffer (2.5 mM imidazole. pH 7.0) supplemented with protease inhibitors (1:100, protease inhibitor cocktail; Sigma Aldrich). Cells were unroofed by mild application of lysis buffer through a 5-ml syringe fitted with a blunted, flattened 18-gauge needle. Periodically, the state of unroofing was observed in cells by inverted phase-contrast microscopy. Footprints were then fixed in 2% formaldehyde in PBS for 5 minutes and permeabilized with 0.1% TritonX-100 in PBS (wash buffer). After wash, cells were immunostained with phospho-PKCδ (S643 PKCδ/S676 PKC) at 1:50 dilution in 30 mg of BSA. Secondary antibody conjugated to Alexa-594 was utilized to immunostain for 1 hour. Nuclei were stained with DAPI in PBS. 

## Supporting Information

Movie S1 **Mapping of PKCδ to force exertion.** GFP-linked PKCδ CaaX stably transfected cells were induced with FBS and force exerted on the substratum was calculated and false-colored red, whereas the PKC is false-colored white. Shown is a representative cell at 10 minute intervals for 80 minutes.
(AVI)

Movie S2 **Membrane-targeted PKCδ exert increased protrusion.** (2)PKCδ CaaX/ (3)SaaX stably transfected cells were induced with 5 nM EGF and 5 nM insulin as described in (Figures 3a, 4b). Images were taken at 20X objective magnification with resolution 0.35um/pixel. Movie frames were at 15 minute intervals for 5 hours.
(AVI)

Movie S3 **Membrane-targeted PKCδ exert increased protrusion.** (2)PKCδ CaaX/ (3)SaaX stably transfected cells were induced with 5 nM EGF and 5 nM insulin as described in (Figures 3a, 4b). Images were taken at 20X objective magnification with resolution 0.35um/pixel. Movie frames were at 15 minute intervals for 5 hours.
(AVI)
